# Case Report: Surgery and genetic analysis of a complete androgen insensitivity syndrome family with testicular malignant tumors

**DOI:** 10.3389/fgene.2023.1048600

**Published:** 2023-03-21

**Authors:** Lu Jiang, Peng Jia, Baofeng Duan, Yan Zhang

**Affiliations:** Department of Obstetrics and Gynecology, Peking University First Hospital, Beijing, China

**Keywords:** complete androgen insensitivity syndrome, seminoma, AR gene, whole exome sequencing, case report

## Abstract

**Introduction:** Complete androgen insensitivity syndrome (CAIS) is a rare sex development disorder that results from X-linked androgen receptor gene mutations. Malignant transformation of the gonads is the most feared complication in postpubertal patients.

**Methods:** In the current report, primary amenorrhea, infertility, and groin mass were symptoms described by a 58-year-old woman and his younger sister. Their two aunts, who shared the same clinical traits, passed away for an unknown reason.

**Results:** After gonadectomy, both patients were diagnosed with seminoma and an extratesticular benign tumor, and the elder sister suffered from breast cancer about a year after the operation. The diagnosis of CAIS was verified by whole-exome sequencing (WES), in which an uncommon mutation (c.2197G>A) in the AR gene was identified.

**Discussion:** This is the first family report of CAIS with germ cell tumors. The identified AR gene mutation based on WES can expand the understanding of CAIS.

## Introduction

Androgen insensitivity syndrome (AIS) is the most prevalent disease of sex development, with a karyotype of 46, XY and a predicted incidence of 1:20,000–1:100,000 (Tyutyusheva et al., 2021). Complete androgen insensitivity syndrome (CAIS) typically manifests as a female phenotype in a person with an XY karyotype and testes producing androgen whose levels are normal for their age, which is caused by X-linked androgen receptor (AR) gene mutations (Hughes et al., 2012; Mongan et al., 2015). In light of the effect of anti-Müllerian hormone (AMH), which is created by Sertoli cells of the testis, the uterus, cervix, and proximal vagina are absent in CAIS. Gonads are frequently discovered in the lower abdomen or in the inguinal canals, where they can lead to bilateral inguinal hernias or labial edema. The abundant conversion of androgens to estrogens by the P450 aromatase enzyme during puberty causes the spontaneous development of breast and female obesity along with a typical growth spurt. However, the vagina was a blind bottom, and there was hardly any pubic or axillary hair. Because of the presence of the Y chromosome, women with CAIS are typically taller than average (Hughes et al., 2012; Coutifaris et al., 2018). The most concerning consequence for women with CAIS is malignant alteration of the gonads, while the risk is positively correlated with age (Tyutyusheva et al., 2021). In this article, we present two individuals with CAIS and pathologically confirmed testicular seminoma from the same family. After whole-exome sequencing (WES), a rare AR gene mutation site (c.2197 G > A) was detected. The discovery of this study can expand the understanding of CAIS, especially the pathogenesis on a molecular scale.

## Case description

**FIGURE 1:**
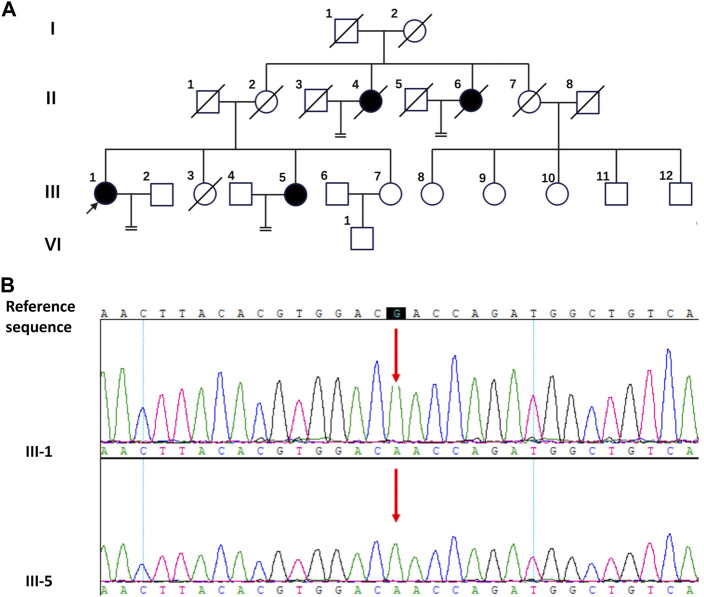
The pedigree diagram of the Chinese family with CAIS and the result of Sanger sequencing. **(A)** The pedigree diagram of the Chinese family. Black arrow indicates the proband, III-1; shapes in black indicate affected individuals, II-4, II-6, III-1and III-5. **(B)** Analysis of the DNA sequence. Sanger sequencing show the sequence encompassing the missense mutation (c.2197G>A) in the AR gene in the proband (III-1) and his sister (III-5).

**FIGURE 2:**
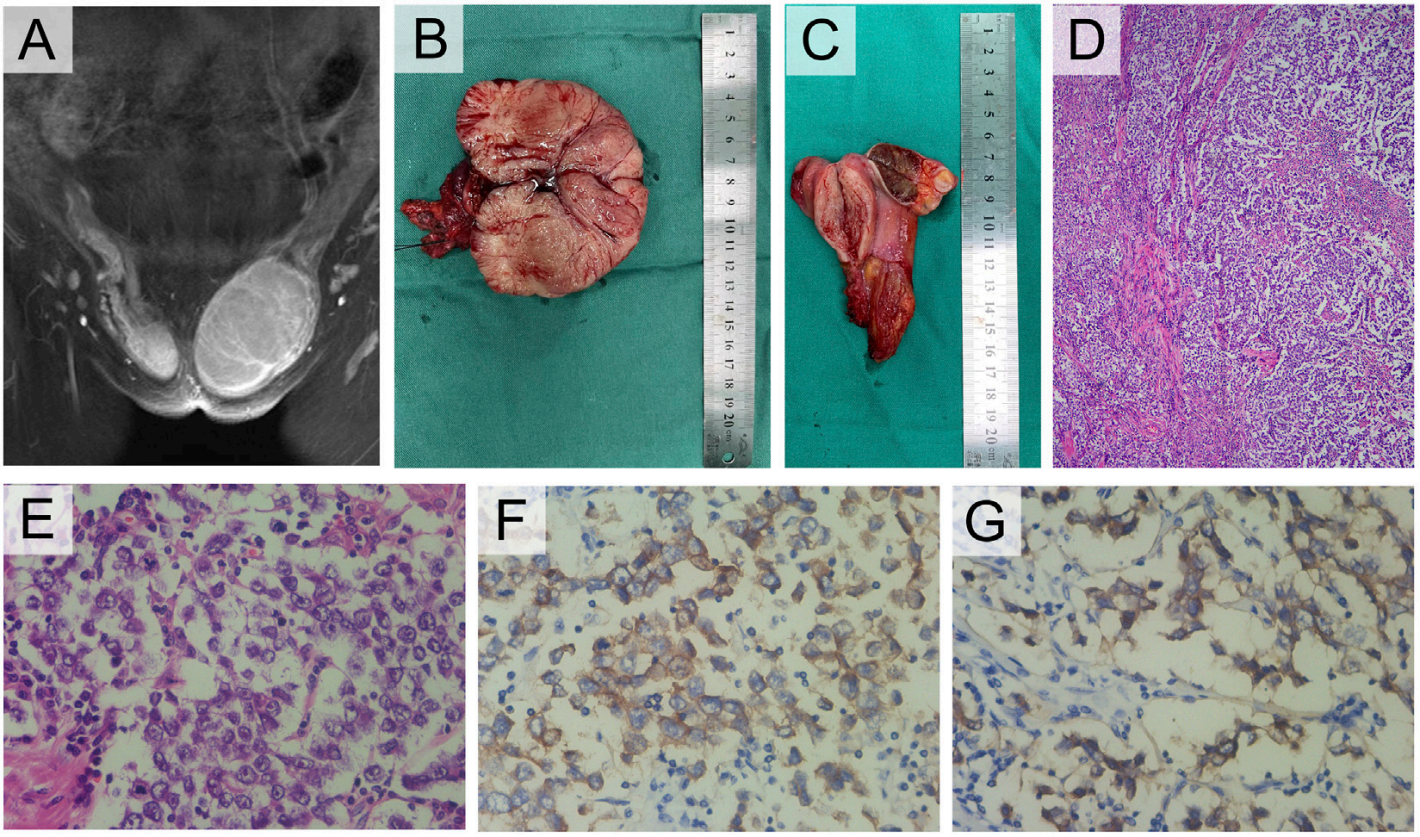
Clinical and pathological manifestations of III-1. **(A)** Enhanced MRI of groin mass. **(B)** Photograph of the left gross pathology specimen showed a lobulated fleshy mass. **(C)** The right specimen showed a brown cross section similar to testis and a muscular mass nearby. **(D)** Photomicrograph showed a sheetlike arrangement of tumor cells separated by fibrous septa. **(E)** The tumor cells exhibited cytoplasmic clearing and squared-off large nuclei (H-E stain, 400×). **(F)** The tumor cells displayed cytoplasmic expression of PLAP (IHC, 400×), and **(G)** cell membrane-localized expression of CD117 (IHC, 400×).

In the studied family, a 58-year-old woman served as the proband (III-1), who was admitted to our hospital complaining of primary amenorrhea and increased inguinal mass for half a year. The patient stated that she has gone untreated for inguinal edema since she was a baby. She had normal sex after marriage despite having primary amenorrhea and infertility. The left inguinal mass dramatically grew to 10 cm in the past half a year, which prompted her to seek a medical assessment. Examination revealed that the person was 85 kg and 168 cm tall. The patient’s pubic and axillary hair was sparse. A pelvic examination revealed normal female external genitalia, and the vagina was a blind channel with normal length (approximately 6 cm) and rugae. The cervix and uterus were absent. The left inguinal mass was approximately 10 cm × 7 cm in size, while the right side was 3 cm in diameter. Bilateral inguinal masses were inactive, without tenderness. The remainder of the physical examination was normal. Pelvic enhanced MR indicated that the uterus and ovary were absent, and there were mixed signal masses in the bilateral inguinal canal with irregular enhancement (Figure 2A). Laboratory sex hormone test results are shown in Table 1, which suggested a significantly elevated testosterone level of 4.55 ng/mL. The results of tumor markers (Alpha-fetoprotein (AFP), Lactate dehydrogenase (LDH), human chorionic gonadotrophin (hCG)) were normal, and the results of peripheral blood karyotype analysis showed 46, XY. In the patient’s lineage, two aunts (II-4 and II-6) and one younger sister (III-5) shared the proband’s primary amenorrhea and infertility. Unfortunately, the first and second generations perished, and the cause was unclear. The phenotypes of her two sisters (III-3 and III-7) were normal, and III-3 died by accident (Figure 1A).

**TABLE 1:** Changes in hormone levels before and after surgery of III-1.

Hormones	Before operation	After operation	References range	
		(D4)	Postmenopausal women	Men
LH (mIU/mL)	13.02	19.47	10.87–58.6	1.24–8.62
FSH (mIU/mL)	22.81	27.59	16.7–113.6	1.27–19.26
E2 (pg/mL)	31.00	16.40	0–38.9	0–38.95
PRL (ng/mL)	10.62	19.32	3.34–26.72	2.64–13.13
T (ng/mL)	4.55	0.42	0.10–0.75	1.75–7.81

LH, luteinizing hormone; FSH, follicle-stimulating hormone; E2, estradiol; PRL, prolactin; T, testosterone.

The patient had hernia sac high ligation and bilateral inguinal mass ectomy after being admitted. The general findings of the specimen showed that the left inguinal mass was well circumscribed, fleshy, bulging and hard, with a size of 10 cm × 7 cm × 6 cm, yellowish white in cross section (Figure 2B). The right mass was brown in cross section similar to testis, beside which a muscular tissue with a diameter of approximately 3 cm can be found (Figure 2C). The pathological results confirmed seminoma in the left testis, which did not invade the tunica albuginea, stage pT1, while the right mass was cryptorchidism with angioleiomyoma of the spermatic cord. The immunohistochemistry (IHC) staining results of the left inguinal mass were placental alkaline phosphatase (PLAP) ++, CD117 +++, AE1/AE3 -, AFP -, and HCG - (Figures 2D, E, F, G). The final diagnosis was left testicular seminoma (stage IA, pT1N0M0S0), and radiotherapy was offered 1 month after the operation. Notably, on the fourth day after the operation, the testosterone level decreased significantly (Table 1). The patient reported she was diagnosed with breast cancer 11 months after the surgery and underwent a breast-conserving lumpectomy during the most recent follow-up.

**FIGURE 3:**
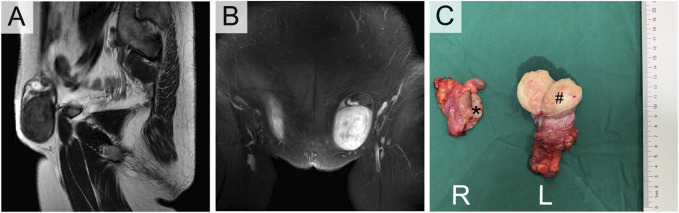
Enhanced MRI **(A,B)** and Orchiectomy specimens **(C)** of III-5. *Right testicular seminoma, # Left paratesticular leiomyoma.

Her sister (III-5) was 49 years old with clinical manifestations basically consistent with those of III-1. She had primary amenorrhea and infertility, and her left inguinal mass was significantly enlarged, with a diameter of approximately 5 cm, in the past 2 years. Physical examination revealed that her weight was 74 kg with a height of 168 cm. Pelvic examination and enhanced MR indicated that the uterus and ovary were absent. The left inguinal mass was approximately 5 cm in diameter, while the right inguinal mass was 3 cm. Enhanced MR revealed a circular mixed low signal mass in both groins (Figures 3A, B). Laboratory tests suggested an elevated testosterone level of 5.12 ng/mL. The results of tumor markers (AFP, LDH, hCG) were also normal, with a peripheral blood karyotype analysis showing 46, XY. She underwent the same operation as her sister, and the final diagnosis was right testicular seminoma (stage IA, pT1N0M0S0) and left paratesticular leiomyoma. Interestingly, the leiomyoma appeared to originate from the tunica vaginalis of the left testis. Her surgical specimen photos are shown in Figure 3C.

For III-1 and III-5, WES investigation of mutations utilizing peripheral blood was carried out in addition to clinical evaluations, which were conducted by BGI Clinical Laboratories Co., Ltd. (Shenzhen, China). A sample of the patients’ blood was used to extract the DNA, and 20,000 exons as well as the mitochondrial genome were sequenced. Sequence alignment using BWA was performed on the reference genome UCSU hg19. ExomeDepth was used to identify exon-level copy number alterations. The sequencing depth of 99.95% of the loci was over 20 × average depth. This study’s most significant mutation was found in the protein-coding region of exon five of the AR gene on the X chromosome (c.2197G>A, p. Asp733Asn), which Sanger sequencing verified (Figure 1B). The c.2197G>A mutation of the AR gene has been reported in previous studies (Chaudhry et al., 2017; Cools et al., 2017). It was found in a 1-month-old female infant and a 16-year-old CAIS patient. The female infant was diagnosed with germ cell neoplasia *in situ* (GCNIS) after gonadectomy. A missense mutation (c.2197G>A) in the AR gene may be the cause of the observed pathogenicity of CAIS in this lineage when the aforementioned conditions are combined.

## Discussion

The term “androgen insensitivity syndrome” refers to a group of illnesses where a person with an XY karyotype can produce normal levels of androgen but exhibits complete or partial resistance to the biological effects of androgen due to mutations in the X-linked androgen receptor gene, which codes for the ligand-activated androgen receptor, a transcription factor and member of the nuclear receptor superfamily. According to different biological phenotypes, it is usually divided into three subtypes: complete androgen insensitivity syndrome (CAIS) characterized by the feminization of the testes, partial androgen insensitivity syndrome (PAIS) with external genitalia more masculine and mild form androgen insensitivity syndrome (MAIS) with only breast development and infertility. Among CAIS patients, primary amenorrhea in adolescence or inguinal swelling in a neonate are the two most common presentations (Hughes et al., 2012). Proband III-1 and her sister III-5 in the current family were compatible with the diagnosis of CAIS when combined with clinical signs and auxiliary examinations. First, both of them presented with an inguinal mass, primary amenorrhea and infertility, scant pubic and axillary hair and typical female external genitalia. Second, imaging revealed that the uterus and ovaries were absent, and hormone tests showed that testosterone was in line with the level of men of the same age. Finally, additional genetic testing revealed that both patients had karyotypes of 46, XY. WES sequencing showed that both of them carried the same missense mutation in the AR gene (c.2197G>A).

The 920 amino acid AR protein is divided into eight exons (listed as 1–8) and seven introns, with a molecular mass of 110 kDa. The single-stranded polypeptide AR receptor has four major structural domains. The ligand-binding domain (LBD, amino acids 646–920), encoded by exons 4–8, contains particular androgen binding sites, various transcription factors of coactivation and the activation function-2 (AF-2) region. It encourages the receptor’s engagement with cytoplasmic heat shock proteins (HSPs), which is followed by the androgen hormone and results in the migration of the AR into the nucleus (Tyutyusheva et al., 2021). According to the HGMD database (human gene mutation database, http://www.hgmd.cf.ac.uk/ac/gene.php?gene=AR), there are 618 types of AR gene mutations, of which 443 are missense/non-sense mutations. The mutation c.2197G>A (p. Asp733Asn) found in the present study is a single nucleotide missense mutation with few previous reports. In addition to the two CAIS patients mentioned above (Chaudhry et al., 2017; Cools et al., 2017), this mutation site is also included in the DSD-related panel designed by Eggers et al. (2016). On the current basis, according to the standard of ACMG guidelines (2015) (Richards et al., 2015), this mutation site is likely pathogenic, and there is a lack of evidence of strong pathogenicity. After adding the families reported in this study, the evidence of pathogenicity of this mutation will be further strengthened.

The likelihood of gonadal malignancies developing in DSD individuals increases when Y chromosomal material is present in the gonadal karyotype and the gonad is located in the abdominal or inguinal region. The probability of gonadal tumors is lowest (5%) in patients with CAIS and highest (15%–60%) in those with 46, XY gonadal dysgenesis, while the rate of tumor incidence is 15% in patient with PAIS(9, 10). Age has been recognized as a significant risk factor for the development of gonadotrophic tumors. Deans et al. (2012) found that the probability of developing a neoplasm is approximately 0.02% in women under 30 and up to 22% in those over that age. Puberty testicles can provide sex hormones necessary for growth and development in children with CAIS. However, to avoid the malignant transformation of cryptorchidism, gonadectomy is usually recommended after puberty, followed by hormone replacement therapy (Hughes et al., 2012; Mongan et al., 2015). Chaudhry et al. (2017) included 133 patients with CAIS who received gonadectomy. The incidence of malignant tumors was only 1.5% (2/133). Two patients underwent gonadectomy at the ages of 30 and 68, and postoperative pathology suggested seminoma and malignant sex cord-stromal tumor, respectively. Other benign gonadal tumors included Sertoli cell adenoma, testicular hamartoma and GCNIS. GCNIS, the premalignant precursor of germ-cell tumors, is hypothesized to result from a developmental arrest of embryonic germ cells and develops from gonocytes or primordial germ cells. If gonadectomy is not performed, there will be an increasing possibility of malignant germ-cell tumors, including seminoma, non-seminoma and dysgerminoma, with increasing age (Hughes et al., 2012). Even more rarely, sex cord–stromal tumors may develop in patients with AIS, which are most commonly Sertoli-cell adenomas (Coutifaris et al., 2018). Gonadectomy after puberty should continue to be the first-line recommendation to reduce the potential increased risk of cancer. For females who want to maintain their gonads, Döhnert et al. (2017) recommended a routine yearly or biannual screening program that included endocrine assessment, gonadal imaging and the measurement of certain tumor markers (AFP, hCG, LDH, and optionally PLAP in non-smokers). In addition, preventive gonadectomy is also controversial. The prevalence rate of pre-GCNIS is 10%–15% in patients with CAIS and PAIS aged from 14 to 54 years, only a few of these diseases will progress to malignant experimental germ cell tumors, and the genetic susceptibility of different individuals may lead to diverse disease outcomes (Cools et al., 2017).

In the present study, III-1 and III-5 underwent gonadectomy at the ages of 58 and 49, respectively, and were both diagnosed with testicular seminoma. To the best of our knowledge, this is the first family to include 2 or more CAIS patients complicated with malignant germ cell tumors. Because of some social factors, both of them existed in their past lives as normal women. Although they cannot have children and menstruation, they do not want to reveal the secrets of their family until they are worried about the obvious increase in inguinal tumors. Fortunately, both of the sisters presented with stage I seminoma, with a survival rate of 99% (Döhnert et al., 2017). Considering the large size of the tumor, adjuvant radiotherapy was offered postoperatively.

In addition to testicular malignancies, it is worth mentioning that benign tumors of paratesticular tissues were found in both patients in the present study, including spermatic cord angioleiomyoma in III-1 and paratesticular leiomyoma in III-5. First, the spermatic cord is rarely the site of malignancies, between which lipomas are the most prevalent benign tumor, whereas sarcomas are the most common malignant type. Only one case of angioleiomyoma of the spermatic cord has been reported to date (Ghei et al., 2005). In addition, paratesticular leiomyoma is another rare tumor of the male reproductive system. To the best of our knowledge, there have been only four reports of paratesticular leiomyoma (Krichen Makni et al., 2005; Goulis et al., 2006; Gorunova et al., 2011; Savaş-Erdeve et al., 2016). In the only case of a normal male phenotype, der(14)t(12;14)(q15;q24) was found, as reported in uterine leiomyomas (Gorunova et al., 2011). The other three patients all suffered from sex development disorder, including a 29-year-old girl with testicular feminization syndrome and two girls with CAIS, aged 17 and 18, respectively. Leiomyomas are typically very rare tumors of the male urogenital tract that can develop either intratesticularly or paratesticularly. The origin of these tumors is thought to be the seminiferous tubules and paratesticular structures, as well as the smooth muscle cells of the interstitial stroma (Savaş-Erdeve et al., 2016). As we mentioned above, aromatase in peripheral tissues of CAIS patients can convert androgen into estrogen. The high estrogen status of these patients may be a trigger for paratesticular leiomyoma. It is significant to note that prior to this case, there had not been any reports of breast cancer developing later in life, despite the fact that women with CAIS had sustained estrogen exposure (Mongan et al., 2015). This is the first case report of CAIS complicated by breast cancer and seminoma occurring simultaneously.

Psychosocial support is another key issue that should be considered after surgery. They can still return to their original life and work roles as women after gonadectomy. Support from their families and partners is the most important factor. Furthermore, doctors should disclose as little information about their gender development abnormalities to others as possible and give appropriate hormone replacement therapy when necessary to improve their quality of life.

In summary, this work describes an uncommon mutation in the AR gene, c.2197G>A, in a family with CAIS and an accompanying seminoma. This is the first report of multiple cases of germ cell tumors in the CAIS family. In addition, the coexistence of spermatic cord angioleiomyoma, paratesticular leiomyoma and breast cancer, as in our case, seems to be an extremely rare finding. The assessment of connections between CAIS genotypes and phenotypes, particularly tumor risk, is facilitated by the reporting of these CAIS-associated mutations, aiding future genetic consultation and diagnosis. (Abacı et al., 2015; De Toni et al., 2019).

## Data Availability

The datasets presented in this study can be found in online repositories. The names of the repository/repositories and accession number(s) can be found below: https://www.ncbi.nlm.nih.gov/, PRJNA940202.

## Abbreviations

CAIS, complete androgen insensitivity syndrome; AR, androgen receptor; WES, whole-exome sequencing; AMH, anti-Müllerian hormone; IHC, immunohistochemistry; GCNIS, germ cell neoplasia *in situ*; PAIS, partial androgen insensitivity syndrome; MAIS, mild form androgen insensitivity syndrome; LBD, ligand-binding domain; DSD, disorders of sex development; AFP, alpha-fetoprotein; LDH, lactate dehydrogenase; hCG, human chorionic gonadotrophin.
